# Relationship of ankyloglossia and obstructive sleep apnea: systematic review and meta-analysis

**DOI:** 10.1007/s11325-024-03021-4

**Published:** 2024-03-13

**Authors:** Sara Camañes-Gonzalvo, José María Montiel-Company, Vanessa Paredes-Gallardo, Francisco Javier Puertas-Cuesta, Rocío Marco-Pitarch, Marina García-Selva, Carlos Bellot-Arcís, María Dolores Casaña-Ruiz

**Affiliations:** 1 Sleep Unit. Department of Stomatology, Faculty of Medicine and Dentistry, University of Valencia, Valencia, Spain; 2 Senior Lecturer. Department of Stomatology, Faculty of Medicine and Dentistry, University of Valencia, Valencia, Spain; 3 Sleep Unit, Faculty of Medicine and Health Sciences, Catholic University of Valencia San Vicente Mártir, Valencia, Valencia Spain; 4 Department of Stomatology, Faculty of Medicine and Dentistry, University of Valencia, Valencia, Spain

**Keywords:** Ankyloglossia, Short lingual frenulum, Obstructive sleep apnea, Sleep-disordered breathing; Pediatric dentistry

## Abstract

**Purpose:**

Recent studies have highlighted the potential role of a short lingual frenulum as a risk factor for pediatric obstructive sleep apnea syndrome. A shortened frenulum may contribute to abnormal orofacial development, leading to increased upper airway resistance and susceptibility to upper airway collapsibility during sleep. Recognizing early indicators, such as a short lingual frenulum, is crucial for prompt intervention. This systematic review aims to evaluate the association between a short lingual frenulum and the risk of obstructive sleep apnea syndrome in children.

**Methods:**

This systematic review adheres to PRISMA criteria for a quantitative analysis. A comprehensive search was conducted on five databases until January 2024 to identify relevant studies. The selected articles underwent rigorous analysis, considering study design, sample characteristics, lingual frenulum characterization, sleep assessment methods, and key findings.

**Results:**

A total of 239 references were initially identified. Finally, six studies were included in the qualitative synthesis, with four studies eligible for the quantitative synthesis. The Newcastle–Ottawa scale was employed to assess study quality. Meta-analysis, supported by a moderate evidence profile according to the GRADE scale, revealed statistically significant differences, with odds ratios of 3.051 (confidence interval: 1.939 to 4.801) for a short frenulum and 12.304 (confidence interval: 6.141 to 24.653) for a high-arched palate.

**Conclusion:**

This systematic review and meta-analysis provide evidence supporting the association between ankyloglossia and obstructive sleep apnea in children. Nevertheless, it is crucial to consider additional factors such as tongue mobility and the presence of a high-arched palate in further evaluations.

**Supplementary Information:**

The online version contains supplementary material available at 10.1007/s11325-024-03021-4.

## Introduction

Obstructive sleep apnea syndrome (OSAS) stands as a prevalent form of sleep-disordered breathing (SDB), marked by the partial or complete obstruction of the upper airways, resulting in compromised ventilation. Its etiology is intricate, necessitating personalized treatment approaches [1]***.*** This condition is estimated to impact approximately 1–4% of children, with the highest incidence occurring between 2 and 8 years of age [2].

Children with OSAS are prone to experiencing severe neurobehavioral issues, including hyperactivity, daytime sleepiness, attention disorders, cognitive function decline, and learning difficulties [3]. Moreover, OSAS can induce fundamental alterations in pharyngeal muscle tone and reflex responses, along with metabolic disturbances and cardiovascular anomalies [4]*.* Indeed, recent studies have reported a possible influence of calcium (Ca), Magnesium (Mg), vitamin D, and auric acid concentrations on the sleep architecture of patients with arterial hypertension (AH) and comorbid OSA. Moreover, alterations in Mg concentration were observed among hypertensive individuals with OSA. [5]

The repercussions of sleep-disordered breathing extend beyond the individual, posing significant public health implications. Despite its incidence rising with age, recent research suggests that many individuals, both children and adults affected by OSAS, remain undiagnosed and consequently untreated [6].

Several risk factors contributing to OSAS in children have been identified, including obesity, adenoid and tonsil hypertrophy, allergic rhinitis, septal deviation, and, more recently, a short lingual frenulum [7]*.* Emerging evidence suggests that a brief lingual frenulum may contribute to OSAS development in children, as the tongue, a pivotal muscle group in the upper airways, plays a crucial role in maintaining oropharyngeal patency [8].

Tongue tie, a relatively rare congenital anomaly, manifests in approximately 4–5% of the general population. It follows an autosomal dominant inheritance pattern linked to the X chromosome, with a higher prevalence in males than females. The phenotypic spectrum ranges from the absence of clinical significance to complete ankyloglossia, where the ventral part of the tongue merges with the floor of the mouth [9].

Assessment of the lingual frenulum can be conducted through direct measurement, Kotlow's free tongue measurement, or by evaluating tongue mobility. Direct frenulum measurement involves gauging the distance between the lingual frenulum insertion and the tongue, but studies have shown its inferiority compared to tongue mobility assessment due to technical challenges and a higher risk of measurement errors. Conversely, measuring tongue mobility is a relatively straightforward parameter that can be completed quickly. Notably, prior investigations into the association between tongue tie and obstructive sleep apnea (OSA) often focused solely on tongue free length without considering tongue mobility [10, 11].

Over the last few decades, research has unveiled the negative consequences of short tongue tie in children, including complications with breastfeeding, speech impediments, and challenges in performing mechanical and social skills such as lip licking and maintaining oral hygiene. Ankyloglossia is speculated to alter tongue position, leading to maxillary underdevelopment, mandibular prognathism, and malocclusions like crossbite or open bite. Moreover, a shortened tongue tie has been associated with oral breathing, abnormal oral cavity development, and an elevated risk of upper airway collapsibility [12]*.*

At birth, the tongue naturally rests in the upper part of the palate, and its continuous engagement in sucking, swallowing, and chewing stimulates the intermaxillary synchondrosis—an active process until 13–15 years of age—contributing to normal oral-facial growth. This physiological tongue position aligns with normal nasal breathing, emphasizing the intricate relationship between tongue activity and facial development [13].

Conversely, a lack of bone-to-bone interaction, limited growth stimulation, and nasal breathing issues can disrupt orofacial development. This disruption may lead to a reduction in the ideal size of the upper airway, potentially causing abnormal breathing patterns during sleep. This initial limitation of airflow could progress over time, resulting in a gradual worsening of obstructive events, eventually culminating in obstructive sleep apnea.

There exists compelling evidence linking the presence of a shortened lingual frenulum to alterations in craniofacial growth, with potential implications for upper airway caliber. The primary objective of the current meta-analysis is to establish a definitive relationship between ankyloglossia and obstructive sleep apnea. The null hypothesis determines no association between the presence of a shortened lingual frenulum and the occurrence of obstructive sleep apnea.

Although sleep apnea in adults is increasingly better understood, there remains a significant amount of misunderstanding regarding children. Given the systemic consequences of a short lingual frenulum during pediatric years, it is crucial to understand these adverse outcomes and their association with potential breathing-related sleep disorders.

## Materials and methods

This systematic review followed the 2020 PRISMA (Preferred Reporting Items for Systematic Reviews and Meta-Analyses) guidelines [14]. The current systematic review had been pre-registered on PROSPERO with the registration number CRD42022375113.

### PICO question

The primary aim was to address the PICO (Population/Patient, Intervention, Comparison, Outcome) question: Is there a relationship between ankyloglossia and sleep-disordered breathing in children?

### Inclusion and exclusion criteria

The research comprised "Articles" and "Articles in Press," encompassing randomized clinical trials, longitudinal studies, retrospective and prospective cohort or case–control studies. No limitations were imposed regarding the publication year or language. Inclusion criteria involved investigations focusing on children diagnosed with sleep-related breathing disorders through polysomnography (PSG) and aged between 4 and 17 years. Exclusion criteria encompassed children with a positive history of acute or chronic cardiorespiratory or neuromuscular diseases, chronic inflammatory diseases, major craniofacial abnormalities, chromosomal syndromes, and epilepsy.

### Search strategy

An extensive electronic search was conducted across major databases, including Medline (PubMed), Excerpta Medica Database (EMBASE), Scopus, Web of Science, and the Cochrane databases, to identify potentially relevant studies, regardless of language barriers. Gray literature was explored using Opengrey. In specific cases, direct communication via email was established with the respective authors to obtain any crucial information not available in the published articles. Furthermore, a meticulous manual review of the references cited in the included studies was undertaken to identify any pertinent articles meeting the inclusion criteria that may not have been retrieved by the electronic databases. The search strategy was last revised in January 2024.

### Search terms

The search strategy utilized an extensive set of keywords, including "obstructive sleep apnea," "snoring," "sleep-related breathing disorder," "ankyloglossia," "lingual frenulum," and "short lingual frenulum." Boolean operators ("OR" and "AND") were employed to connect terms relevant to the research question (refer to Table 1 in the Online Resource).

The specified keywords were organized into three groups, and a thorough search, encompassing all potential combinations within these groups, was conducted. For data integrity, the located articles were imported into Mendeley Desktop 1.13.3 software (Mendeley Ltd, London, England) to identify and eliminate duplicates. The detailed search strategy for all databases is provided in the supplementary materials.

### Selection process

Two reviewers working independently (SC-G and MDC-R) methodically reviewed the titles and abstracts of all identified articles. Any disagreements were resolved through consultation with a third reviewer (RM-P). In instances where abstracts did not provide adequate information for a definitive decision, the reviewers moved on to a comprehensive analysis of the full-text articles. During the subsequent stage of study selection, the same evaluators conducted a thorough scrutiny of the full-text articles, documenting the rationale for exclusion in accordance with pre-established inclusion and exclusion criteria.

### Study data

For each article, the meticulous recording encompassed key variables such as author and publication year, study type, geographic location, study population characteristics, reference guide, sample size, demographic variables (gender and age), severity of obstructive sleep apnea (OSA), and the assessment method. These main variables were further categorized into clinical variables (age, gender, short frenulum, high arch palate, and free tongue length) and diagnostic variables (risk of OSA evaluation method—such as Pediatric Sleep Questionnaire (PSQ), Home Sleep Cardio-Respiratory Studies (HSCS) or OSA questionnaire for children; medical examination method—encompassing height and weight, BMI; and ankyloglossia evaluation method—utilizing tools like the Quick Tongue-Tie Assessment Tool).

### Risk of bias/ Quality assessment

The evaluation of study quality was independently carried out by the identical investigators, employing the Newcastle–Ottawa scale for observational studies [15]. Disagreements among the investigators were resolved through consensus, seeking the input of a third investigator in cases of uncertainty. Assessment criteria, including study design, risk of bias, result consistency, indirect evidence, imprecision, and publication bias [16], were utilized to evaluate the overall strength of evidence through the grading of recommendations, assessment, development, and evaluations tool (GRADE Handbook).

### Summary measures and synthesis approach

Odds ratios (OR) with corresponding 95% confidence intervals (CI) were calculated to assess the risk of obstructive sleep apnea (OSA) for patient gender (males), short frenulum, and high arch palate. Additionally, mean differences with 95% CI were computed for the variable of free tongue length (mm).

### Statistical analysis

The synthesized data from the studies were integrated into a random-effects model using the inverse variance method. Heterogeneity among the studies was evaluated through the Q test and the I2 statistic. A Q test p-value < 0.1 was indicative of significant heterogeneity. To explore potential publication bias, funnel plots, Egger’s regression intercept method, and the classic fail-safe number were employed. All statistical analyses were conducted using Comprehensive Meta-Analysis V 3.0 Biostat software.

## Results

**Fig. 1:**
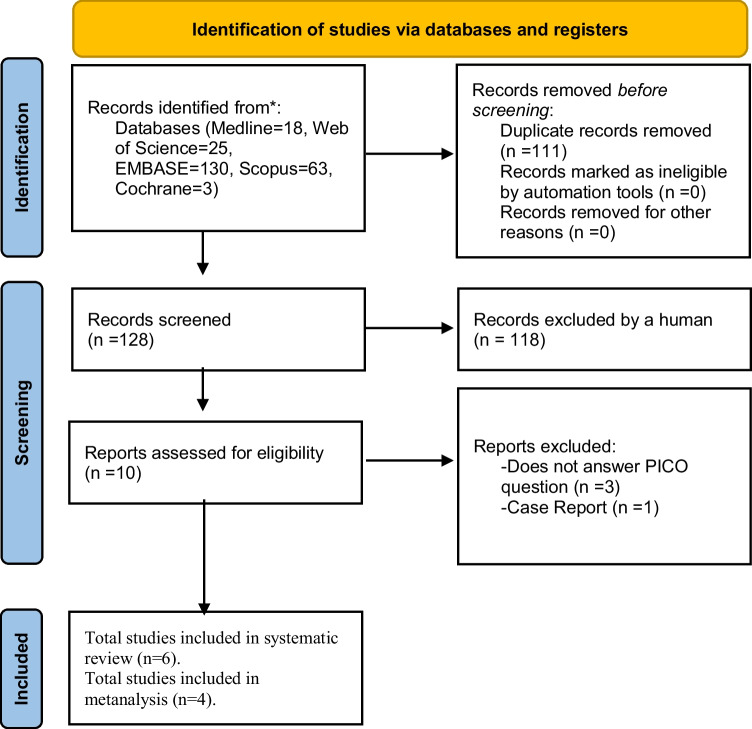
PRISMA 2020 flow diagram for new systematic reviews which included searches of databases and registers only

The initial search across various databases yielded 239 references relevant to the PICO question—18 in PubMed, 63 in Scopus, 130 in EMBASE, and 25 in Web of Science. After eliminating 111 duplicated articles, the remaining 128 underwent initial scrutiny. Subsequently, based on the examination of titles and abstracts, 118 articles were excluded as they did not align with the research question. Thorough reviews of the full texts of the resulting 10 articles led to the exclusion of four. In the end, six articles that satisfied the eligibility criteria underwent qualitative analysis, whereas four underwent quantitative analysis. The detailed overview of the article selection process is illustrated in the PRISMA flowchart, as shown in Fig. 1 (Fig. 1).

### Characteristics of the included studies

Table 1 summarizes the key characteristics of the six studies incorporated into the systematic review. Sample sizes across the selected articles varied, ranging from a minimum of 69 [11] to a maximum of 504 [8], with the majority falling within the range of 130–150 patients. Regarding gender distribution, most studies demonstrated a balanced representation, such as in [17] with 66 men vs. 69 women or [18] with 67 men vs. 64 women. The average patient age in the studies hovered around 9 years, indicating a predominant focus on younger children.

**Table 1:** Characteristics of the studies included in the systematic review

Study	Study type	N (case/control)	Years (case/control)	Male (case/control)	OSA diagnosis
Burska Z et al. 2022 [17]	CC	131(65/66)	3–17 (9.5 ± 3.0 /9.5 ± 3.0)	(64/0)	PSQ
Yuen H et al. 2022 [18]	CS	82(48/34)	5–12 (8.4 ± 1.7/8.2 ± 1.7)	(36/21)	PSG
Brozek—Mądry E et al. 2021 [16]	CC	135(67/68)	4 –17 (9.4 ± _3.0/9.5 ± 3.1)	(48/50)	PSQ
Cohen-Levy J et al. 2020 [10]	C	69(69/0)	4- 17 (9.3 ± 3.6 /0)	(42/0)	HSCS HSAT PSG SCR
Villa M et al. 2020 [7]	C	504(42/462)	9.6 (10.2 ± 3.1/10.1 ± 2.0)	(199/23)	SCR
Guilleminault C et al. 2016 [19]	C	150 (63/87)	(9.88 ± 3.21 /8.05 ± 3.59)	(63/87)	PSQ

CC: case–control, CS: Cross-sectional, C: Cohort. N: Number of patients. SCR: Sleep Clinical Record; PSQ: Pediatric sleep questionnaire; HSCS: Hierarchic severity clinical scale; HSAT: Home sleep apnea test

All included studies followed an observational design [8, 11, 17–20]. Among them, one was a cross-sectional study [20] (82 patients included)], two were case–control studies [18, 19] (266 patients included) and three were cohort studies [11, 20] (723 patients included).

### Quality assessment

All included articles demonstrated high quality on the Newcastle–Ottawa scale (Table 2, 3 and 4 Online Resource), exhibiting a low risk of bias across a minimum of 17 out of the 22 evaluated items. Both significant associations presented a moderate evidence profile as determined by the GRADE Handbook (Table 5 Online Resource).

### Qualitative synthesis

#### Risk of OSAS evaluation method

Five studies employed the Pediatric Sleep Questionnaire (PSQ) as the primary method for assessing the risk of obstructive sleep apnea (OSAS) [11, 17–20]. Noteworthy for its high specificity and sensitivity in detecting OSA risk in children, the PSQ is advantageous due to its minimal reliance on medical staff involvement [21]. Additionally, other studies utilized the Hierarchic Severity Clinical Scale (HSCS) to assess self-reported sleep-disordered breathing (SDB) symptoms [11, 22], while some conducted a sleep apnea test following a positive result on an OSA questionnaire for children [11, 20].

#### Medical examination method

All studies incorporated a comprehensive physical examination and medical history assessment. Physical examinations involved measurements of height, weight, and BMI [8, 11, 17, 19], evaluation of the oral cavity for the presence of a high-arched palate [8, 11, 17–20], Mallampati classification of the oropharynx [11, 18, 20] assessment of palatine tonsil size [18–20], occlusion [8, 11, 18], and the evaluation of anthropometric and craniofacial parameters, such as the length of the free tongue [17, 19, 20], length mobility [8, 11, 19, 20], and inter-incisor distance [17, 20].

#### Ankyloglossia evaluation method

Regarding ankyloglossia evaluation, most studies utilized the commercially available caliper Quick Tongue-Tie Assessment Tool, measuring inter-incisor distance and the length of the free tongue [8, 17–20], following the methods of Kotlow et al. [23, 24] or Ruffoli et al. [10]. Cohen-Levy J. et al. considered ankyloglossia as a limited tongue evaluation in relation to a maximal aperture of 60% or less [11].

### Quantitative synthesis

**Fig. 2:**
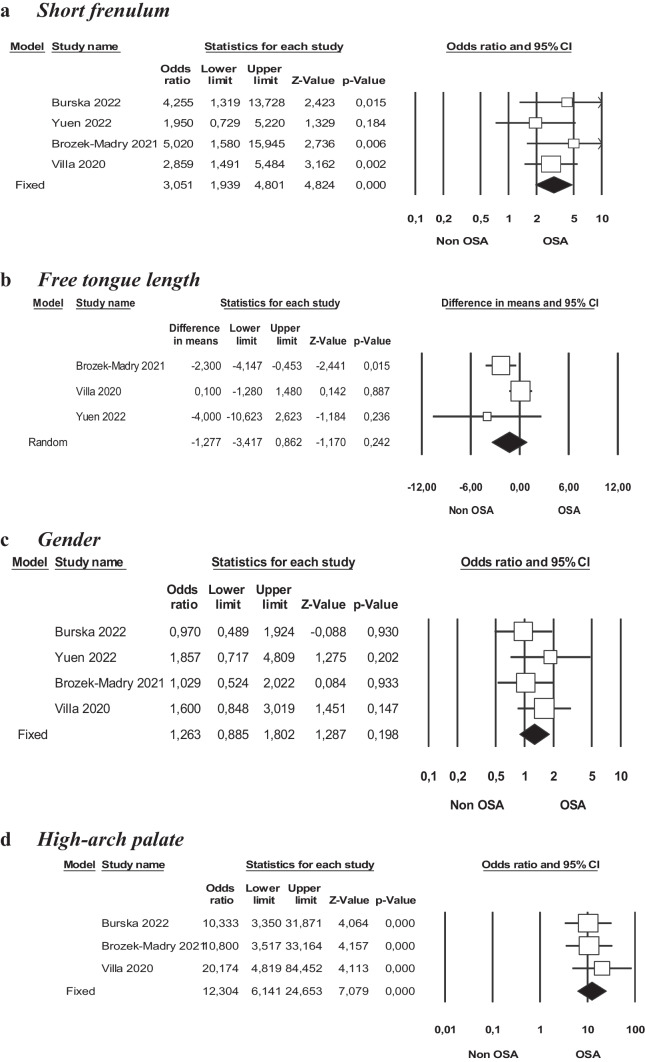
Forest Plot of meta-analysis

Figure 2a-d, presents the results of the quantitative analysis. In relation to short frenulum (Fig. 2a) and high arch palate (Fig. 2d), both exhibited a significant association with obstructive sleep apnea (Table 2), as determined by the GRADE tool, indicating a moderate evidence profile. The forest plot of this meta-analysis depicted odds ratios of 3.051 and 12.304, with 95% confidence intervals ranging from 1.939 to 4.801 and 6.141 to 24.653, respectively. Moreover, the I^2^ value (I^2^ = 0.000%) suggested an absence of heterogeneity (Q = 1.855; p < 0.603; Q = 0.602; p < 0.740).

**Table 2:** Meta – analysis results. Odds ratio of risk factors and event rate

Variable	Q-value	P-valor* (Q)	I^2^%	OR	CI
Short frenulum	1.855	0.603	0.000	3.051	[1.939–4.801]
Hight arch palate	0.602	0.740	0.000	12.304	[6.141–24.653]
Gender (males)	2.088	0.554	0.000	1.263	[0.885–1.802]

^*^p < 0.05. Abbreviations. I^2^: heterogeneity; OR: Odds ratio; CI: confidence interval

The calculated associations for gender (males) and free tongue length did not reveal statistically significant relations with short frenulum or obstructive sleep apnea (Table 2 and 3).

**Table 3:** Meta – analysis results. Mean difference of free tongue length

Variable	Q-value	P-value*	I^2^%	Point estimate	CI	P-value*
Free tongue length (mm)	5.058	0.080	60.45	-1.277	[-3.417–0.862]	0.242

^*^p < 0.05. Abbreviations. I^2^: heterogeneity; CI: confidence interval

#### Publication bias

**Fig. 3:**
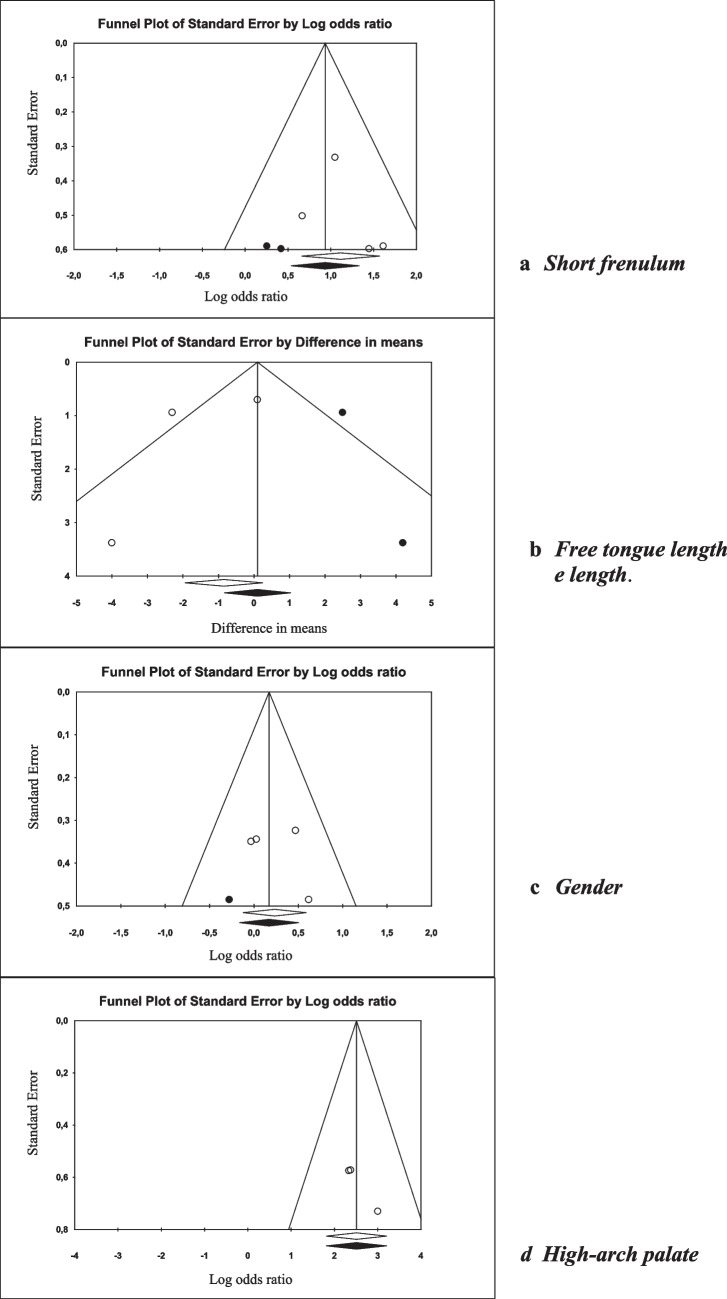
Funnel Plot of meta-analysis

The funnel plots exhibited symmetrical images, and no disparities in estimations were observed when imputed values were added. The symmetry in the plot for this variable strongly indicates the absence of publication bias (Fig. 3).

Furthermore, the Egger's regression intercepts calculated for the various estimations did not demonstrate statistical significance (Table 4).

**Table 4:** Egger’s regression intercept, Duval and Tweedie’s and Classic fail-safe n-studies

Variable	Egger’s regression intercept			Duval and Tweedie’s		Classic fail safe–N studies
	Egger intercept	Egger IC	P-value*	To left	To right	
Gender (males)	2.12	[-11.81—16.04]	0.05	1.19 [0.85–1.66]	*	-
Short frenulum	1.24	[-6.08—8.55]	0.54	2.55 [1.71–3.79]	*	21
High arch palate	4.11	[-0.34—8.57]	0.054	*	*	37
Free tongue length	-1.841	[-29.87—26.19]	0.56	*	0.10 [-2.15–2.35]	-

^*^p < 0.05. Abbreviations. N: number; CI: confidence interval

## Discussion

Pediatric sleep apnea, a subtype of sleep-disordered breathing (SDB), is a prevalent condition with potential implications for a child's health and development [4]. It is important to note that other pathologies, such as sleep bruxism, temporomandibular disorders, and dental caries, can also be associated with sleep disorders [25]. Emerging research suggests that a short lingual frenulum may contribute to the onset of obstructive sleep apnea syndrome (OSAS) in children, highlighting the multifaceted nature of its etiology [8, 11, 17–20]. Ankyloglossia, characterized by a restrictive lingual frenulum, has been associated with breastfeeding difficulties, including challenges such as poor latching, inability to maintain latch, poor weight gain, irritability during feeding, and maternal pain [9].

Recent data from the United States reveal a noteworthy 110.4% increase in reported diagnoses of ankyloglossia in newborns from 2012 to 2016, as indicated by Wei EX et al.'s comprehensive review of various databases [25]. Meanwhile, the prevalence of OSA in children has been reported to range from 1 to 5% [26].

Given the high prevalence of ankyloglossia and OSAS, both of which carry potential long-term health consequences for children, this review aims to systematically evaluate the evidence surrounding the relationship between ankyloglossia and OSAS. Contrary to the null hypothesis, our analysis of various orofacial variables revealed significant associations with short frenulum and high arch palate, while other analyzed variables did not exhibit statistically significant effects.

The variables under scrutiny in this systematic review fall into two distinct categories: clinical and diagnostic. In the realm of obstructive sleep apnea syndrome (OSAS) diagnosis, polysomnography (PSG) stands as the gold standard. However, this method is known for its expense, limited accessibility, and potential stress for children. Cohen-Levy et al. and Guilleminault et al. advocate a judicious approach, suggesting that PSG should be reserved for children identified as at risk through questionnaires [11, 20].

Cohen-Levy J et al. specifically employed the Hierarchic Severity Clinical Scale (HSCS) to evaluate sleep-disordered breathing (SDB) symptoms. Participants reporting chronic snoring (HSCS > 0) or scoring positive for suspected OSA (HSCS > 2.72), in the absence of craniofacial syndrome, underwent a sleep apnea test. In contrast, other authors, such as those using the Pediatric Sleep Questionnaire (PSQ), diagnosed OSA without recourse to a sleep apnea test and, consequently, without the involvement of medical staff. The current guidelines from the Working Group of the European Respiratory Society endorse the PSQ as a screening tool for detecting OSA risk in children [2, 27]. Notably, the PSQ boasts the highest specificity among all available questionnaires for children, minimizing the occurrence of false positive results. Authors of the PSQ recommend that eight or more "YES" answers in the questionnaire qualify a child for inclusion in the OSAS risk group [21].

In the evaluation of sleep-disordered breathing (SDB) and obstructive sleep apnea syndrome (OSAS), Villa M et al. and Cohen-Levy J et al. utilized the Sleep Clinical Record (SCR), a comprehensive clinical tool designed to identify children at high risk for SDB. The SCR calculates a total score, considering various factors, including abnormalities in the nose, oropharynx, dental and craniofacial occlusion, the Brouillette OSAS score [28], and the presence of symptoms related to inattention and hyperactivity. A total SCR score of 6.5 is deemed positive, indicating a high risk of OSAS defined by an obstructive apnea–hypopnea index (AHI) > 1 episode/h [11, 29].

Regarding the diagnosis of ankyloglossia, the Quick Tongue-Tie Assessment Tool was predominantly employed across studies. This tool measures the inter-incisive distance with the tongue in a low-placed position and the tip of the tongue against the palate. It calculates the percentage difference between measurements, considering normal if the difference is < 50%. Additionally, the tool measures the distance from the insertion of the frenulum at the base of the tongue to the tip of the tongue [8, 17, 19, 20].

Two distinct measurement methods were identified in the literature. The Kotlow method, utilized by Villa M et al., Brozek-Madry et al., Burska Z et al., and Yuen H et al., measures the "free tongue," defined as the length from the insertion of the lingual frenum into the base of the tongue to the tip of the tongue. A normal frenulum length is considered > 16 mm [8, 17–19]. In contrast, the Ruffoli method measures the length of the frenulum itself, classifying a normal frenulum length in children aged ≥ 6 years as > 20 mm, with a mild problem at 16–19 mm [10]. Guilleminault et al. [20] considered > 16 mm as a cut-off point for a normal free-tongue length for children aged ≥ 3 years [23].

Tongue characteristics play a crucial role in assessing the risk of obstructive sleep apnea (OSA). Some studies, such as those by Guilleminault C (2016) and Yoon A (2017), conducted a systematic examination using maneuvers defined by myofunctional therapists. This evaluation included assessing the placement of the tongue at rest, the capability of performing maneuvers like touching the nose and chin with the tip of the tongue, creating a "cigar tongue" while protruding the tongue, touching the median raphe with the tip of the tongue with a wide-open mouth, and observing upper jaw closure during the maneuver. Additionally, the ability to pronounce certain letters and vowels was considered.

Yuen H et al. defined tongue mobility as Mpal/Mmax, representing the maximal distance between incisors when the tongue tip touched the palatal papilla and during full mouth opening, respectively, obtained using a digital caliper [30]. Low tongue mobility was defined as mobility less than 60% [11, 19]. Interestingly, trials such as the one by Fioravanti M (2021) demonstrated that diode laser lingual frenectomy therapy can improve the severity of OSAS in pediatric patients [31]. Similarly, lingual frenuloplasty with myofunctional therapy, as indicated by Zaghi S et al. (2019), is deemed safe and potentially effective. These findings underscore the importance of an early diagnosis and treatment of ankyloglossia, coupled with myofunctional therapy, in pediatric subjects experiencing sleep apnea problems. Such interventions showcase the potential for targeted therapeutic approaches to mitigate OSA severity and improve overall outcomes in affected children [32].

Considering the clinical variables in pediatric SDB, the literature consistently describes a male predominance and younger age at surgery in pediatric SDB, possibly attributed to a less mature craniofacial skeleton and sexual dimorphism or android pattern of fat distribution in older adolescents. However, the present meta-analysis on the "gender" variable did not reveal significant differences between genders. This outcome might be explained by the fact that OSA becomes more pronounced in males after puberty [33].

As regards “short frenulum” variable, the meta-analysis results demonstrated an association between a shortened lingual frenulum and SDB. Specifically, ankyloglossia appear in OSA patients 3.05 times more than in non-OSA patients. Ankyloglossia limits the upward movement of the tongue, thus preventing the formation of lip seal during swallowing, leading to tongue thrusting. Abnormal bone growth stimulation, an absence of nasal breathing (due to anatomical and muscle tone dysfunction) with secondary development of mouth breathing are responsible for the abnormal oral-facial bone structures supporting the upper airway, thus increasing the risk of UA collapse during sleep [34].

Concerning the variable of a "short frenulum," the outcomes of the meta-analysis revealed a notable correlation between a shortened lingual frenulum and SDB. More specifically, ankyloglossia manifests in patients with OSA at a rate 3.05 times higher than in non-OSA patients. Ankyloglossia restricts the superior movement of the tongue, thereby impeding the establishment of a lip seal during the act of swallowing, consequently inducing tongue thrusting. This phenomenon contributes to abnormal stimulation of bone growth, coupled with an absence of nasal breathing (attributed to anatomical and muscle tone dysfunction), resulting in secondary development of mouth breathing. This, in turn, contributes to the anomalous development of oral-facial bone structures that support the upper airway, thereby elevating the susceptibility to Upper Airway (UA) collapse during the sleep cycle [35].

Furthermore, diminished tongue mobility has been linked to constriction of the maxillary arch [36]. The findings from this meta-analysis underscore a connection between a high-arched palate and SDB. Specifically, the presence of a palate characterized by both height and narrowness correlated with a 12.3-fold increase in the risk of SDB. A narrow maxilla combined with a high-arched palate is associated with heightened nasal airflow resistance and posterior displacement of the tongue [37]. Palatal assessment in pediatric populations is a straightforward procedure that should be an integral component of SDB screening protocols in children.

Evaluation of a high-arched palate was conducted by Burska Z et al., Brozek-Madry E et al., Cohen-Levy J et al., Villa M et al., and Guilleminault C et al., solely utilizing a subjective evaluation method (YES/NO). A high-arched palate was assessed based on its curvature, characterized by an abnormally pronounced curvature angled superiorly along the palatal midline [8, 11, 17, 20].

In concordance with the findings of Bussi M (2021) [38], this review substantiates a noteworthy association between lingual frenulum alteration and Obstructive Sleep Apnea. A notable limitation of this systematic review is the modest sample size across the included studies, with the absence of an adequate sample size justification. These characteristics potentially introduce a bias in extrapolating the results beyond the confines of the study. One plausible explanation for the challenge of attaining larger sample sizes in pediatric OSA studies is the existence of accessibility barriers to PSG examinations, encompassing high costs and increased wait times for public health services. Consequently, there is a limitation in the diagnostic methodology, given that polysomnographic examination stands as the gold standard for detecting sleep apnea, yet not all authors have uniformly employed this diagnostic modality. Notably, many children refused to undergo sleep studies or decline additional tests after an initial failure. It is imperative to establish a standardized diagnostic protocol inclusive of the evaluation of tongue mobility and palate anatomy during pediatric dentistry and orthodontics appointments. Following a positive outcome in this evaluation and other diagnostic tests, a sleep study should be recommended in accordance with the international consensus on sleep apnea in children [38].

OSA in children has been correlated with various comorbidities and disorders, including respiratory complications, obesity, adenotonsillar hypertrophy, and craniofacial and behavioral syndromes. The studies included in this review reported the exclusion or matching of participants based on factors such as obesity, craniofacial syndromes, and adenotonsillar size, with a majority of them affirming the non-influence of these features on their results. Integration of the BMI variable into the meta-analysis was precluded due to the lack of homogeneity in reporting results. Discrepancies in BMI calculation methodologies were observed across articles, wherein some computed BMI as body weight divided by the squared height, while others utilized BMI z-scores, representing deviations from the mean adjusted for age and sex.

It would be intriguing to investigate whether there exists a correlation between surgical treatment of the frenulum and the amelioration of sleep apnea in children. According to the literature, surgery proves to be more effective than myofunctional therapy, in order to enhances tongue mobility, strength, and endurance, alleviates sleep apnea, reduces mouth breathing and snoring, improves quality of life, decreases teeth clenching, addresses myofascial tension, alleviates post-surgery pain, and enhances speech sound production. However, further studies are necessary to confirm this hypothesis [39, 40].

The outcomes of this systematic review and meta-analysis underscore a noteworthy correlation between ankyloglossia and pediatric obstructive sleep apnea, as indicated by a moderate evidence profile according to the GRADE tool. Nevertheless, it is imperative to recognize that the assessment of a short lingual frenulum should not be regarded in isolation. A comprehensive evaluation should extend to encompass additional factors, notably the assessment of tongue mobility and the presence of a high-arched palate. Therefore, future investigations ought to prioritize the examination of obstructive sleep apnea improvement concerning interventions such as frenectomy and myofunctional therapy.

### Supplementary Information

Below is the link to the electronic supplementary material.

Supplementary file1 (PDF 416 KB)

## Data Availability

Data available upon reasonable request in line with relevant restrictions, e.g. privacy or ethical.

## Declarations

### Protocol and registration

The present systematic review was previously registered in PROSPERO under registration number: CRD42022375113.
