# Supplementary material for: Relationship of ankyloglossia and obstructive sleep apnea: systematic review and meta-analysis
Source: Sleep Breath. 2024 Mar 13;28(3):1067–78. doi: 10.1007/s11325-024-03021-4 (PMC11196303; doi:10.1007/s11325-024-03021-4)
Supplement: Supplementary file 1 — Supplementary file1 (PDF 416 KB) [file 11325_2024_3021_MOESM1_ESM.pdf]

Relationship of ankyloglossia and obstructive sleep apnea: systematic review and meta-analysis.

Sleep and Breathing

Sara Camañes-Gonzalvo; José María Montiel-Company; Vanessa Paredes-Gallardo; Javier Puertas-Cuesta; Rocío Marco-Pitarch; Marina García-Selva; Carlos Bellot-Arcís; Maria Dolores Casaña-Ruiz.

*Corresponding author:

José María Montiel-Company.

Faculty of Medicine and Dentistry. University of Valencia. C/Gascó Oliag 1 – 46010 Valencia, Spain.

Email: jose.maria.montiel@uv.es

Online Resource

Table 1. Electronic search strategy for the different databases.

**PubMed (MEDLINE)**	
Item	Search strategy
Total	(ankyloglossia OR "lingual frenulum" OR "short lingual frenulum") AND ("obstructive sleep apnea" OR "sleep apnea" OR "sleep-disordered breathing").
**Web of science**	
Item	Search strategy
Total	(ALL=(Ankyloglossia OR "lingual frenulum" OR "short lingual frenulum")) AND ALL=("obstructive sleep apnea" OR "sleep apnea" OR "sleep-disordered breathing").

**EMBASE**	
Item	Search strategy
Total	('ankyloglossia'/exp OR ankyloglossia OR 'lingual frenulum'/exp OR 'lingual frenulum' OR 'short lingual frenulum') AND ('obstructive sleep apnea'/exp OR 'obstructive sleep apnea') OR 'sleep apnea'/exp OR 'sleep apnea') AND ('pediatric dentistry'/exp OR 'pediatric dentistry').
**SCOPUS**	
Item	Search strategy
Total	(ALL (ankyloglossia OR "lingual frenulum" OR "short lingual frenulum")) AND (ALL("obstructive sleep apnea" OR "sleep apnea" OR "sleep-disordered breathing")) AND (ALL ("pediatric dentistry")).
**COCHRANE**	
Search strategy	
Total	(Ankyloglossia OR “lingual frenulum” OR “short lingual frenulum”) AND (“obstructive sleep apnea” OR “sleep apnea” OR “sleep-disordered breathing”).

Table 2. Quality of cohort studies assessed using the Newcastle-Ottawa Quality Assessment Form for Cohort Studies.

	Selection														Comparability			Outcome										
**AUTHOR.YEAR**	1a	1b	1c	1d	2a	2b	2c	3a	3b	3c	3d	3e	4a	4b	1a	1b	1c	1a	1b	1c	1d	1e	2a	2b	3a	3b	3c	3d
**Cohen-Levy J. et al. 2020**	*				*								*		*	*	*						*			*		
**Guilleminault C. et al. 2016**	*				*								*		*	*	*						*			*		
**Villa MP. et al. 2020**	*				*								*		*	*	*						*			*		

Items: Selection 1) Representativeness of the exposed cohort: a) Truly representative; b) Somewhat representative; c) Selected group; d) No description of the derivation of the cohort. 2) Selection of the non-exposed cohort: a) Drawn from the same community as the exposed cohort; b) Drawn from a different source; c) No description of the derivation of the non-exposed cohort. 3) Ascertainment of exposure: a) Secure record (e.g., surgical record) (one star); b) Structured interview (one star); c) Written self-report; d) No description; e) Other. 4) Demonstration that outcome of interest was not present at start of study :a) Yes ;b) No. Comparability : 1) Comparability of cohorts on the basis of the design or analysis controlled for confounders : a) The study controls for age, sex and marital status ; b) Study controls for other factors (list) ; c) Cohorts are not comparable on the basis of the design or analysis controlled for confounders . Outcome: 1) Assessment of outcome: a) Independent blind assessment; b) Record linkage; c) Self report; d) No description; e) Other. 2) Was follow-up long enough for outcomes to occur: a) Yes; b) No. Indicate the median duration of follow-up and a brief rationale for the assessment above. 3) Adequacy of follow-up of cohorts: a) Complete follow up- all subject accounted for; b) Subjects lost to follow up unlikely to introduce bias- number lost less than or equal to 20% or description of those lost suggested no different from those followed. (one star); c) Follow up rate less than 80% and no description of those lost; d) No statement.

Table 3. Quality of cohort studies assessed using the Newcastle-Ottawa Quality Assessment adapted for cross sectional studies.

	Selection											Comparability		Outcome					
**AUTHOR.YEAR**	1a	1b	1c	1d	2a	2b	3a	3b	4a	4b	4c	1a	1b	1a	1b	1c	1d	2a	2b
**Yuen HM. et al. 2022**	*	*			*		*				*	*				*		*	
**Brozek-Madry E. et al. 2021**	*	*			*		*				*	*				*		*	

Items: Selection: 1) Representativeness of the cases: a) Truly representative of the HCC patients (consecutive or random sampling of cases). b) Somewhat representative of the average in the HCC patients (non-random sampling).c) Selected demographic group of users. 0 score; d) No description of the sampling strategy. 0 score, 2) Sample size: a) Justified and satisfactory (≥ 400 HCC included). 1 score; b) Not justified (<400 HCC patients included). 0 score 3) Non-Response rate: a) The response rate is satisfactory (≥95%). 1 Score; b) The response rate is unsatisfactory (<95%), or no description.4) Ascertainment of the screening/surveillance tool: a) Validated screening/surveillance tool. 2 scores; b) Non-validated screening/surveillance tool, but the tool is available or described. c) No description of the measurement tool. 0 score. Comparability: (Maximum 1 stars);1) The potential confounders were investigated by subgroup analysis or multivariable analysis. a) The study investigates potential confounders. 1 score; b) The study does not investigate potential confounders. Outcome: (Maximum 3 stars) 1) Assessment of the outcome: a) Independent blind assessment. 2 scores; b) Record linkage. 2 scores; c) Self report. 1 score; d) No description. 0 score 2) Statistical test: a) The statistical test used to analyze the data is clearly described and appropriate. 1 score; b) The statistical test is not appropriate, not described or incomplete. 0 score

Table 4. Quality of cohort studies assessed using the Newcastle-Ottawa Quality Assessment Form for Case-Control Studies.

	Selection										Comparability			Outcome									
AUTHOR.YEAR	1a	1b	1c	2a	2b	3a	3b	3c	4a	4b	1a	1b	1c	1a	1b	1c	1d	1e	2a	2b	3a	3b	3c
Burska Z. et al. 2022	*			*				*	*		*	*				*	*		*		*		

Selection .1) Is the case definition adequate?: a) Yes, with independent validation (one star); b) Yes, e.g., record linkage or based on self-report; c) No description. 2) Representativeness of the cases: a) Consecutive or obviously representative series of cases (one star). b) Potential for selection biases or not stated 3) Selection of controls: a) Community controls (one star); b) Hospital controls; c) No description .4) Definition of controls: a) No history of disease (endpoint) (one star); b) No description of source. Comparability 1) Comparability of cases and controls on the basis of the design or analysis controlled for confounders: The study controls for age (one star); Study controls for other factors (list). Cohorts are not comparable on the basis of the design or analysis controlled for confounders. Exposure :1) Ascertainment of exposure: a) Secure record (e.g., surgical record) (one star); b) Structured interview where blind to case/control status (one star); c) Interview not blinded to case/control status; d) Written self-report or medical record only; e) No description; 2) Same method of ascertainment for cases and controls: Yes (one star);3) Non-response rate: a) Same rate for both groups (one star); b) Non-respondents described; c) Rate different between cases and controls with no description

Table 5: GRADE evidence profile for ankyloglossia associations from systematic review and meta-analysis of randomized controlled trials and observational studies.

Quality assessment							Summary of findings		Quality
							OR*	CI	
**Nº studies (Nº patients)**	**Design ***	**Study limitations**	**Inconsistency**	**Directness**	**Imprecision**	**Publication bias**			
**Ankyloglossia is associated to OSA**									
4 (852)	O	-	No (I2= 0%)	Direct	No important	No	3.051	[1.939-4.801]	Moderate
**Ankyloglossia is related to high-arched palate**									
3 (770)	O	-	No (I2= 0%)	Direct	No important	No	12.304	[6.141-24.653]	Moderate

**Abbreviations. O: Observational study; OR: Odds Ratio; CI: Coefficient Interval (95%).*

Table 6. Prisma checklist

Section/topic	#	Checklist item	Reported on page #
**TITLE**			
Title	1	Identify the report as a systematic review, meta-analysis, or both.	1
**ABSTRACT**			
Structured summary	2	Provide a structured summary including, as applicable: background; objectives; data sources; study eligibility criteria, participants, and interventions; study appraisal and synthesis methods; results; limitations; conclusions and implications of key findings; systematic review registration number.	Title page
**INTRODUCTION**			
Rationale	3	Describe the rationale for the review in the context of what is already known.	1
Objectives	4	Provide an explicit statement of questions being addressed with reference to participants, interventions, comparisons, outcomes, and study design (PICOS).	1
**METHODS**			
Protocol and registration	5	Indicate if a review protocol exists, if and where it can be accessed (e.g., Web address), and, if available, provide registration information including registration number.	12
Eligibility criteria	6	Specify study characteristics (e.g., PICOS, length of follow-up) and report characteristics (e.g., years considered, language, publication status) used as criteria for eligibility, giving rationale.	2
Information sources	7	Describe all information sources (e.g., databases with dates of coverage, contact with study authors to identify additional studies) in the search and date last searched.	2-3
Search	8	Present full electronic search strategy for at least one database, including any limits used, such that it could be repeated.	Online resource
Study selection	9	State the process for selecting studies (i.e., screening, eligibility, included in systematic review, and, if applicable, included in the meta-analysis).	3
Data collection process	10	Describe method of data extraction from reports (e.g., piloted forms, independently, in duplicate) and any processes for obtaining and confirming data from investigators.	3

Data items	11	List and define all variables for which data were sought (e.g., PICOS, funding sources) and any assumptions and simplifications made.	3
Risk of bias in individual studies	12	Describe methods used for assessing risk of bias of individual studies (including specification of whether this was done at the study or outcome level), and how this information is to be used in any data synthesis.	3
Summary measures	13	State the principal summary measures (e.g., risk ratio, difference in means).	3
Synthesis of results	14	Describe the methods of handling data and combining results of studies, if done, including measures of consistency (e.g., I^2) for each meta-analysis.	3
Risk of bias across studies	15	Specify any assessment of risk of bias that may affect the cumulative evidence (e.g., publication bias, selective reporting within studies).	3
Additional analyses	16	Describe methods of additional analyses (e.g., sensitivity or subgroup analyses, meta-regression), if done, indicating which were pre-specified.	3
**RESULTS**			
Study selection	17	Give numbers of studies screened, assessed for eligibility, and included in the review, with reasons for exclusions at each stage, ideally with a flow diagram.	4
Study characteristics	18	For each study, present characteristics for which data were extracted (e.g., study size, PICOS, follow-up period) and provide the citations.	4-8
Risk of bias within studies	19	Present data on risk of bias of each study and, if available, any outcome level assessment (see item 12).	4-8
Results of individual studies	20	For all outcomes considered (benefits or harms), present, for each study: (a) simple summary data for each intervention group (b) effect estimates and confidence intervals, ideally with a forest plot.	4-8
Synthesis of results	21	Present results of each meta-analysis done, including confidence intervals and measures of consistency.	6-8
Risk of bias across studies	22	Present results of any assessment of risk of bias across studies (see Item 15).	5
Additional analysis	23	Give results of additional analyses, if done (e.g., sensitivity or subgroup analyses, meta-regression [see Item 16]).	6-8
**DISCUSSION**			
Summary of evidence	24	Summarize the main findings including the strength of evidence for each main outcome; consider their relevance to key groups (e.g., healthcare providers, users, and policy makers).	8-9

Limitations	25	Discuss limitations at study and outcome level (e.g., risk of bias), and at review-level (e.g., incomplete retrieval of identified research, reporting bias).	11
Conclusions	26	Provide a general interpretation of the results in the context of other evidence, and implications for future research.	12
**FUNDING**			
Funding	27	Describe sources of funding for the systematic review and other support (e.g., supply of data); role of funders for the systematic review.	Title page
